# Going for gold: engineering success in elite winter sports

**DOI:** 10.1038/s44172-026-00609-4

**Published:** 2026-02-16

## Abstract

Dr. Julian von Schleinitz is a former professional luge athlete and is currently the Head of Tech Excellence and AI Solutions in Financial Services at BMW Group. He also helps develop technology used to elevate the performance of Olympic athletes as part of BMW’s partnership with the German Luge, Skeleton and Bobsleigh Federation. Here, we talk to Dr. von Schleinitz about his transition from elite athlete to data scientist, the unique engineering and regulatory challenges he’s faced, and the future of artificial intelligence in ice track sports.

**Figure:**
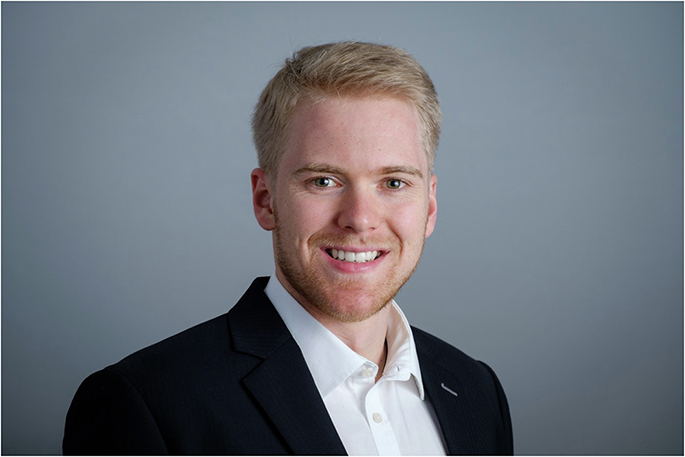
Photograph by Julian von Schleinitz.

1. The Milano Cortina 2026 Winter Olympics is now upon us. Could you tell us about the three different ice track sports that are featuring?

Luge, skeleton and bobsleigh all involve sleds and, at the Olympics, use the same ice track. In luge, athletes ride feet first down the track after an initial push from the start, steering by shifting their weight. Skeleton looks similar to luge, but instead athletes run for around 40 metres before riding head-first and face-down on a sled. Bobsleigh, on the other hand, is often a team sport, with one, two or four people sitting upright in a sled, steering using ropes inside the sled.

All three disciplines are difficult when driving at high speed, although luge is the most specialised and therefore the hardest to learn how to drive down the track safely. Interestingly, you often see athletes that ordinarily compete in summer sports competing in bobsleigh and skeleton, due to the importance of running at the start.

2. Tell us about your background as a professional luge athlete and how you transitioned from participating at the elite level to working on engineering solutions for winter sports?

I started luge when I was 11 years old and joined the German national luge team from 2011 until 2018. I became Junior World Champion and won World Cup medals, but unfortunately never competed in the Olympics as an athlete. I started studying in parallel to luge, at TU Munich and University of Salzburg, graduating with a Bachelor’s in Engineering in 2016 and a Master’s in Chemistry and Physics of Materials in 2017.

In Berchtesgaden, where I come from, we have a strong history of designing and building sleds for winter sports. I worked with famous luge athletes and trainers, including Georg Hackl and Felix Loch, on improving luge sled design. In 2015, conversation started with the BMW Group, technology partner of the German Bobsleigh, Luge and Skeleton Federation, to work closely together on new technologies. Our first project was with BMW Motorsport and their advanced measurement systems. We modified the system to capture the driving dynamics of the luge sled, including longitudinal and lateral speed, acceleration, and yaw rates, which, to our knowledge, had never been done before to that level of detail. As a next step, I developed custom software and algorithms to analyse that data, to help athletes evaluate their performance post-run.

In 2018, I did not qualify to compete with Germany at the Olympics, and retired from luge. At that time, BMW Motorsport were offering a PhD position in data science and AI, and I applied as it appealed to my interests and previous experiences with them. During that PhD, I evaluated race driver performance and developed training simulators for Deutsche Tourenwagen Masters and Formula E. This involved analysing individual driver patterns, preferences and behaviours.

We later transformed this motorsport driving simulator into a bobsleigh simulator, incorporating all the measurement data previously gathered from luge runs. This simulator helped athletes train before the 2022 Winter Olympic Games.

3. What unique engineering challenges have you faced in elite winter sports?

A key challenge we discovered was a lack of directly applicable research and modelling of the physics involved in ice track sports. Motorsport, as a comparison, has a large amount of science research and work behind it. For example, Pacejka models (tyre design models widely used for professional vehicle dynamics simulations) can have over 900 parameters for just one tyre model, including dynamics, forces, load and wear. However, these kinds of physical models are largely non-existent for sled runners.

Another challenge is the lack of understanding of the dynamic physical interactions between the steel that you have in the sled runner and the ice. The underlying physics involves a quasi-liquid layer on the interface between the ice, the air and the runner, and is complex. Furthermore, under the different forces that a sled runner moving at high speed can impose, ice can also melt, shear, and deform.

The literature for ice friction that does exist is also not always applicable to sled-based winter sports. There is a lot of research on minimising the friction coefficient, but it is almost always only for longitudinal friction. In the real world, the friction on a real track is almost 10 times higher than on a perfectly gliding track, because you also have lateral friction from the large (around 110 cm long) blades on luge sleds. On a corner of the track, a runner digs in with the sharp edge of the blade to create friction that causes the sled to turn.

4. How have you worked with new engineering techniques while meeting official sports regulations?

The International Bobsleigh and Skeleton Federation has a rulebook almost 100 pages long, including multiple pages on clothing and footwear. However, working inside restrictions can sometimes lead to creativity. For example, the BMW Group has developed 3D printed spike plates that athletes fit to their own shoes, to give them optimised grip on ice during the initial push phase of a bobsleigh run.

The performance at the start of a bobsleigh run is crucial. The kinetic energy you generate at the start is all you can work with over the whole track – you can only reduce loss in energy. The shoes an athlete wears during this initial run (and the over 250 spikes on the bottom of those shoes) are therefore a key component to optimise. Shoes must be commercially available to meet regulations, but we found many athletes did not like the standard bobsleigh shoes. To solve this, we developed a solution that is within the regulations whilst also benefitting from new technology.

We designed a 3D printed spike plate that athletes could place at the bottom of their own standard tarmac running shoes. We designed the spikes to maximise grip to the ice, experimenting with physical properties and chemical coatings to prevent breaking or wearing. Meanwhile, we ensured they met regulations and including more spikes than necessary in case some break off during use. We used these 3D printed spike plates at the Beijing 2022 Winter Olympics, where Christopher Grotheer won a gold medal for Germany wearing them.

**Figure:**
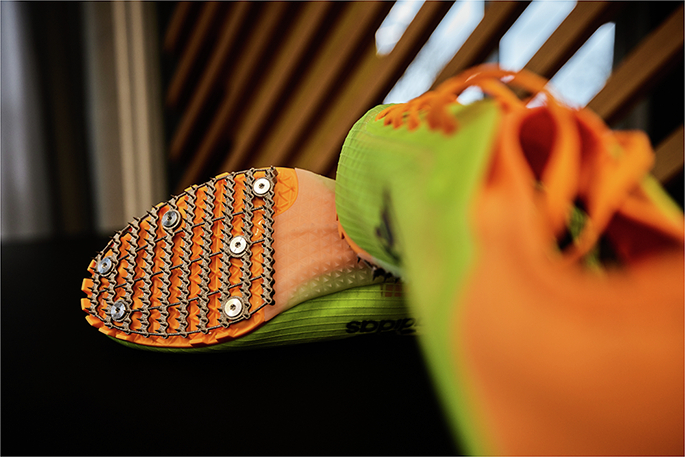
3D printing has enabled athletes to fit custom spike plates to their own shoes, maximising grip to the ice during the start of a luge run while maintaining an athlete’s personal shoe preference. Credit: Photograph from BMW Group PressClub Global. © Copyright BMW AG, Munich (Germany).

5. Have you transferred technology or ideas from motorsport to winter sports?

Yes, this happens often – for example, the motorsport simulator I mentioned earlier that we transformed for bobsleigh runs. We included all the measurement data previously gathered from luge runs, where advanced dynamics sensors were fixed to the sled. We also used the same track mapping technology as used in motorsport to build an accurate 3D representation of the bobsleigh track in Königssee (Germany). This technology can map the track from top to bottom with millimetre accuracy – every bump is correct in the simulated track. The track in Königssee is frequently used by our German athletes for training (although it’s currently under re-construction after a landslide). Because of the familiarity of the track to the athletes, we could adapt the simulator to match how the runners say it should feel, providing a ground truth.

Technology transfer has worked the other way round too. Bobsleigh is quite different from motorsport racing, where you have more or less a two-dimensional track along which the motor vehicles run. In bobsleigh, the track is highly three-dimensional and, in some places, with greater than 90° slope overhangs. There was a simplification in the motorsport simulator that led to problems at those high angles in bobsleigh, which we fixed. But this could also improve accuracy in the motor racetrack model, as racetracks are not strictly 2D – there are some very shallow banks and bumps.

6. You talked about developing simulations of already known tracks, such as the one in Königssee. But what about for new tracks in upcoming competitions?

Of course, we could not perform the same accurate 3D mapping for the track in the Beijing 2022 Winter Olympics. When the track is not yet built, you would need the construction or architectural data to build a simulated track in advance. Host countries do not give away this detailed information – it is in their interest to keep knowledge on track design a secret, to give their own athletes an advantage.

Immediately before the 2022 Olympics, athletes were allowed to perform test runs on the official track, but the Olympic Committee would not have allowed us to scan the track in 3D, as we did in Königssee.

There was, however, an action camera video of a run of the official track, shared by the International Bobsleigh Federation. These action cameras often have a gyroscope and accelerometer built in, and so I wrote an algorithm to extract the driving dynamics data. This data was used in combination with the video material to rebuild the official track in 3D for our simulator. Our team could therefore go to Beijing 2022 already roughly knowing the official track’s layout and intricacies from training with the simulator.

**Figure:**
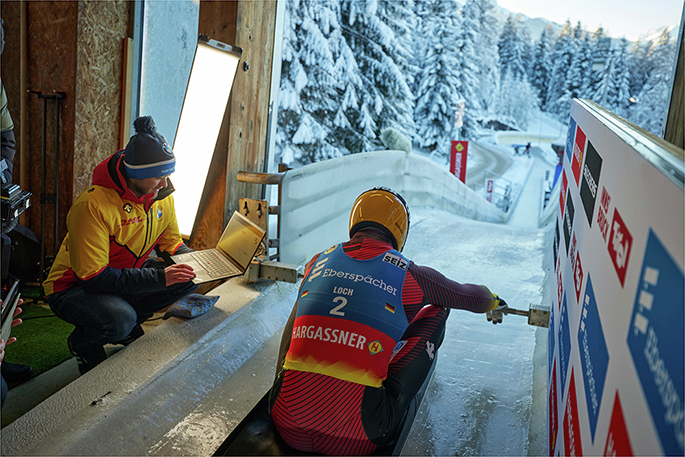
Julian von Schleinitz (left) collecting data from a luge sled fitted with sensors for measuring run dynamics. Credit: Photograph from BMW Group PressClub Global. © Copyright BMW AG, Munich (Germany).

7. Finally, what engineering breakthroughs do you think might fundamentally change winter sports in the next 5 to 10 years?

As a scientist and engineer, it is natural for me to work with data from sports in a technical way. However, for many people, including athletes and trainers, working with data is not natural. AI could be a big chance to give access to data-driven optimisation systems to these kinds of people. For example, an AI agent in the form of a chat bot combined with an expert analytical system could allow athletes to talk to the AI in a natural way to carry out advanced analysis on their performance data.

With luge, bobsleigh and skeleton, you still need a person to go down the track, steer the sled and be brave. But it’s also interesting to see how much performance improves over the years. We see new track records every Olympic cycle, which are being driven by constant improvements in training and technology. For example, Felix Loch is a very successful luge athlete who won gold at the Olympics in 2010 and 2014, and is a strong medal contender for the 2026 Winter Olympic Games. If he didn’t keep up with these technological improvements, he would not even be top 10 anymore. If you are not improving, then you will be overtaken by the competition. You always have to move.

*This interview was conducted by Philip Coatsworth, Associate Editor, and Rosamund Daw, Chief Editor, both from Communications Engineering.*

